# Referral and Utilization of Mental Health Services by Older Adults With Cancer and Factors Influencing Referral From Health Professionals

**DOI:** 10.1002/pon.70573

**Published:** 2026-09-17

**Authors:** Shuman Wang, Maria Ftanou, Lauren Pearson, Chloe Nicholas, Kylie King, Joshua F. Wiley

**Affiliations:** ^1^ Department of Nursing Shanghai Proton and Heavy Ion Center Fudan University Cancer Hospital Shanghai Key Laboratory of Radiation Oncology; and Shanghai Engineering Research Center of Proton and Heavy Ion Radiation Therapy Shanghai China; ^2^ School of Psychological Sciences Turner Institute for Brain and Mental Health Monash University Melbourne Victoria Australia; ^3^ Centre for Mental Health and Community Wellbeing Melbourne School of Population and Global Health The University of Melbourne Melbourne Australia; ^4^ Peter MacCallum Cancer Center Melbourne Victoria Australia

**Keywords:** cancer, mental health services, older, oncology, referral

## Abstract

**Objective:**

Older adults with cancer commonly experience psychological distress. However, the rate at which they receive mental health services is low, and reasons are inconsistently documented. This study aims to characterize adults with cancer referred to mental health services, explore how age influences referrals, and examine factors influencing healthcare professionals' referrals within the Capability, Opportunity, Motivation, and Behavior (COM‐B) model.

**Methods:**

We retrospectively collected data from 557 adults with cancer referred to the Clinical Psychology Department at Peter MacCallum Cancer Center in Melbourne, including demographics, clinical details, and referral information. Additionally, we surveyed 18 healthcare professionals using the General Help‐Seeking Questionnaire, the Gatekeeper Behavior Scale, Mental Health Literacy Scale, and Attitude Questions, which assessed capability (e.g., knowledge), opportunity (e.g., resources), and motivation (e.g., beliefs) in the COM‐B model.

**Results:**

Adults < 65 were more likely to be referred to mental health services than those ≥ 65 (*p* < 0.001), but among those referred there was no significant difference in referral acceptance/uptake rates (*p* = 0.25). Adults < 65 are more likely to be referred for adjustment reasons compared to those ≥ 65 (*p* < 0.05). Healthcare professionals endorse opportunity and motivation factors more when referring adults ≥ 65, but capability factors are a bigger barrier, with fewer than 60% of professionals endorsing nearly half of the capability items.

**Conclusions:**

Older adults with cancer may face inequities in mental health service referrals, but those referred were equally willing to engage. Capability factors present the greatest barrier for professionals referring for mental health, highlighting the need for training.

## 1 Introduction

In 2022, older adults, those aged 65 years and above, accounted for 53% of global new cancer cases and over 60% of cancer‐related deaths [1]. It is estimated that by 2035, the number of new cancer cases among older adults will reach 14 million, accounting for 60% of all new diagnoses [2]. In the dual context of cancer and aging, older adults not only experience the impacts of their existing health conditions but also face socio‐psychological issues, such as over 80% feeling lonely [3], 62.1% reporting psychological distress [4], and 32% exhibiting symptoms of depression [5]. These challenges often result in poorer treatment outcomes, higher healthcare costs, longer hospital stays, and increased risks of suicide and mortality [6, 7, 8, 9]. Therefore, providing timely mental health services for older adults with cancer is crucial for their recovery and survival.

However, up to 75% of older adults with cancer experiencing psychological distress have not accessed mental health services [10]. There are several reasons for this phenomenon. Older adults may refuse mental health services, and they may also be unaware of available services [11, 12]. Despite these barriers, 47% of older adults indicated that they would use mental health services if they knew they were available [13]. Healthcare professionals are the primary point of contact for the older adults seeking mental health services and play a crucial role in referring them to these services [14, 15]. Therefore, whether healthcare professionals proactively refer older adults with cancer may be a key factor affecting their access to mental health services [16]. A 2009 study of 326 patients with metastatic gastrointestinal or lung cancer found that among 76 patients with severe depression, 100% of those under 40 were referred for psychosocial care, while only 22% of those aged 70 and older were referred, indicating that older adults with cancer might indeed be significantly less likely to be referred for mental health services compared to younger individuals [17].

A widely used model to help understand and promote behavior change, the Capability, Opportunity, Motivation, and Behavior (COM‐B) model argues that behavior is influenced by capability (e.g., skills and knowledge), opportunity (e.g., resources and social influences), and motivation (e.g., emotions and beliefs) [18]. The COM‐B model can be applied to understanding the insufficiency of referrals for older adults with cancer. For instance, current research shows that some healthcare professionals struggle to identify mental health problems among older adults with cancer due to a lack of training and knowledge in the context of aging and cancer [16, 19] (reflecting capability), while others report time constraints impacting their ability to identify and manage mental health [16, 19] (reflecting opportunity). Additionally, research has shown some professionals prioritize physical health, believing that mental issues are normal aspects of aging and cancer [20] (reflecting motivation). However, current research has not examined both health professional factors, referrals, and uptake rates for older adults with cancer systematically.

The current study aims to (1) describe the characteristics of adults with cancer referred to mental health services, (2) explore how age influences referrals, reasons, and uptake, and (3) understand the potential factors influencing healthcare professionals' decisions to refer older adults with cancer within the framework of the COM‐B model, and explore where healthcare professionals are more likely to refer them. This is crucial for ensuring equal access to mental health services for older adults with cancer and ultimately improving their mental health and survival outcomes.

## 2 Methods

### 2.1 Study Design

The study was conducted at the Peter MacCallum Cancer Center (Peter Mac) in Melbourne, Australia, and consisted of two stages: a retrospective medical record audit study and a cross‐sectional survey of staff.

### 2.2 Participants and Procedure

#### 2.2.1 Stage 1: Referral Status

We collected retrospective data from the electronic medical record system for individuals with cancer aged 18 and older, referred to the Clinical Psychology Department at Peter Mac over six months between September 1, 2020, and February 28, 2021, without restrictions on gender, cancer type, stage, or treatment. The data includes demographic and mental health referral information. Additionally, clinical information as well as mental health service details were extracted for adults with cancer aged 65 and above (older adults). These referrals came from health professionals at Peter Mac or, rarely, patients self‐referring.

#### 2.2.2 Stage 2: Potential Reasons Influencing Referral Decisions

To better understand factors associated with referral decisions, we surveyed Peter Mac health professionals, the same staff who would have been caring for patients and all referrals (except for rare self‐referrals) in Stage 1. To promote honest responding, the survey was anonymous, making direct linkage with Stage 1 data not possible. Using Peter Mac's staff mailing lists, we emailed healthcare professionals who met the following criteria: (a) working at the Peter Mac with responsibilities allowing for mental health service referrals; (b) aged 18 and above; (c) regularly interacting with older adults. These criteria were assessed by self‐report as part of the consent process. Approximately 1000 staff received this email, which asked them to encourage their colleagues to participate and included a link to the invitation and an explanatory statement. Interested participants completed the survey via Qualtrics after providing their consent. Due to the initial email being sent at the peak of the winter COVID‐19 outbreak, a follow up email was sent the following fortnight to encourage participation.

### 2.3 Measures

#### 2.3.1 Demographic and Clinical Variables

In Stage 1, we retrieved data on age, gender, marital status, language preference, and indigenous status of adults with cancer from the electronic medical record system. For older adults, clinical information was also extracted, including cancer type, treatment modalities, and treatment intent. Long‐term cure or eradication of cancer is considered curative treatment, whereas if medical records indicate that cancer is no longer curable or has progressed to a point where further curative treatment is not feasible, it is considered palliative treatment.

In Stage 2, we conducted a survey to collect information on healthcare professionals' gender, age, profession, years working, role and work setting.

#### 2.3.2 Mental Health Referral‐Related Variables (Stage 1)

We collected information on referrals to the Clinical Psychology Department, including the referrers' discipline, reason for referral, and referral outcome. The referral reasons were extracted from statements made by the referrer regarding why they initiated the referral and categorized into eight major categories: physical‐related (e.g., sleep issues), emotion‐related (e.g. depression, anxiety), cancer recurrence, cancer adaptation (issues related to adjusting to a new diagnosis, prognosis, or terminal diagnosis), treatment‐related (e.g. concerns over treatment decisions), non‐cancer‐related (e.g. interpersonal relationships and COVID‐19 related issues), grief (mourning over the loss of health or things missed due to cancer), and any referrals that do not fit into these categories are classified as “other” (e.g. body image issues). The referral outcome was defined as whether the patient accepted the referral after the intake call from the psychology department.

#### 2.3.3 Mental Health Service‐Related Variables (Stage 1)

Mental health service‐related variables for older adults were extracted, including whether they received an intake call, mental health assessment, mental health care and treatment, support care utilization, and the number of sessions received and the mode of delivery. The above variables are related to a typical referral process to the Outpatient Clinical Psychology Department (details in Supporting Information S1: Figure S1).

In Stage 2, we surveyed healthcare professionals To better understand factors influencing referral decisions. The COM‐B model is a general, theoretical model of behavior, and does not have a standard set of measures used to assess it. Therefore, to assess factors related to mental health referral behaviors in health professionals we selected measures around mental health understanding and behaviors and mapped these conceptually against COM‐B. Where possible we selected commonly used, validated measures. Measures administered in Stage 2 are described below.

#### 2.3.4 Gatekeeper Behavior Scale (GBS; Stage 2)

This 11‐item scale assesses preparedness, likelihood, and confidence in recognizing mental health needs and recommending services [21] (*α* = 0.95 in this study), with responses recorded on a 1–4 or 1–5 Likert scale. Additional relevant items, such as “adequate training,” were included in this study (details in Supporting Information S1: Table S1). Eleven of the thirteen items were used to assess the capability component of the COM‐B model, while two were used to assess the motivation component.

#### 2.3.5 Mental Health Literacy Scale (MHLS; Stage 2)

A subset of 9 items assessed stigma and attitudes toward mental health (*α* = 0.84 in this study), with responses recorded on a 1‐5 Likert scale [22], where a higher score indicates greater mental health literacy (details in Supporting Information S1: Table S2). This was used to evaluate the motivation component of the COM‐B model.

#### 2.3.6 Attitude Questions (Stage 2)

We identified 12 items representing commonly reported barriers to referral derived from existing qualitative literature and organized within the COM‐B framework (details in Supporting Information S1: Table S3) of capability (4 items, e.g. “Mental health problems are a normal part of ageing”), opportunity (3 items, e.g. “I do not have any time to help older patients with their mental health problems”), and motivation (5 items, e.g. “Older patients do not want help with their mental health problems”).

#### 2.3.7 General Help‐Seeking Questionnaire (GHSQ; Stage 2)

This is a 2‐item scale used to assess help‐seeking behaviors and intentions related to mental health, covering twelve domains under each item, with responses recorded on a 1–7 Likert scale. It demonstrates good reliability (*α* = 0.85 in this study) and validity [23]. Additional relevant items, such as “the primary healthcare professionals at Peter Mac,” were included in this study (details in Supporting Information S1: Table S4).

### 2.4 Data Analysis

Analyses were conducted using R 4.4.0. Significance was set at two‐tailed *α* = 0.05.

In Stage 1, descriptive analyses were performed on demographic variables, clinical variables, mental health referral‐related variables, and mental health service‐related variables. A one‐sample proportion test was used to compare the proportion of older adults with cancer referred to the outpatient clinical psychology department with the proportion of older adults with cancer receiving treatment at Peter Mac. Chi‐square tests were used to compare referrer disciplines, reasons for referral, and referral outcomes among different age groups.

In Stage 2, individual items from all questionnaires were categorized as either endorsing or opposing. Coding is detailed in Supporting Information S1: Table S5. Descriptive analyses were conducted on the frequencies and percentages of individual items from all questionnaires, and on the total scale scores for the Gatekeeper Behavior Scale and the Mental Health Literacy Scale. To facilitate the visual display of individual items, we simplified or reversed some of them to ensure that all items had a consistent scoring direction, with higher scores indicating a higher proportion of endorsement. Complete case analysis was used for missing data. Endorsement was defined as selecting the response option indicating a positive response for each item after reverse coding where appropriate, whereas opposing referred to selecting a non‐positive response.

### 2.5 Ethical Approval

Ethical approval was obtained from the Peter MacCallum Cancer Center Human Research Ethics Committee [EC00235] for both Stages 1 and 2, and waived explicit consent requirements for Stage 1.

## 3 Results

### 3.1 Aim 1. Characteristics of Adults With Cancer Referred to Mental Health Services

Between September 1, 2020, and February 28, 2021, inclusive, there were a total of 557 referrals. Detailed demographic information categorized by age is in Supporting Information S1: Table S6. Ages ranged from 21 to 90 years (median = 55.88, SD = 13.36). Of these, 71.6% were patients under 65 years old, 61.8% were female, 60.9% had partners, and 99.3% were non‐indigenous patients.

**FIGURE 1:**
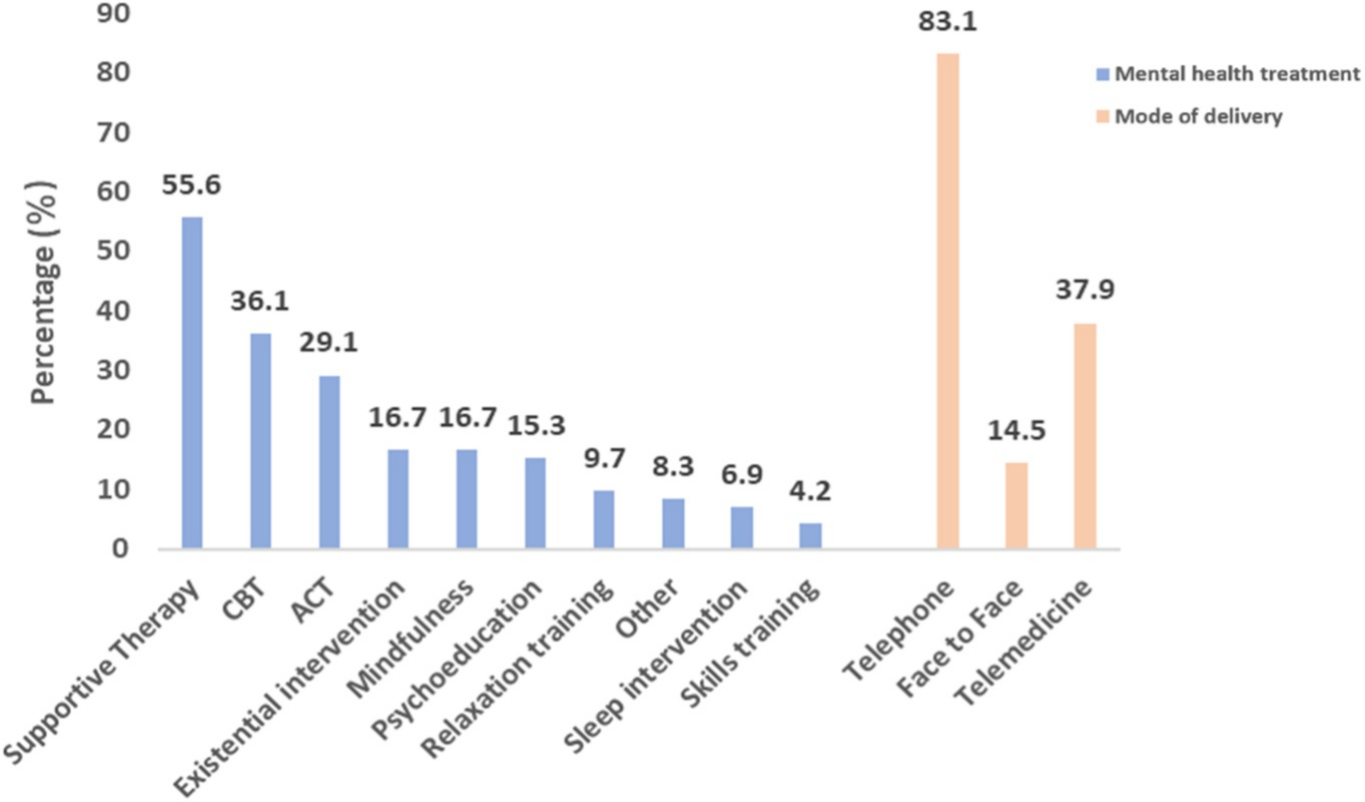
Mental health treatment and mode of delivery. ACT, Acceptance and Commitment Therapy; CBT, Cognitive Behavioral Therapy.

Clinical characteristics and details of mental health services for older adults with cancer are in Supporting Information S1: Table S7. The most common cancer types were gastrointestinal (17.7%), blood (16.5%), and head and neck (15.8%), with 81% undergoing curative treatment. Chemotherapy (33.5%), radiotherapy (29.7%), and surgery (29.1%) were the three most common treatments. Of referred patients, 83.5% received an intake call from the psychology department (with the main reasons for not receiving a call including no response or follow‐up by a psychiatrist instead of a psychologist), 48.7% underwent mental health assessment, and 44.9% received mental health care. Figure 1 shows the specific mental health treatment and psychotherapy mode administered to those who received mental health care. The most commonly received therapies were supportive therapy (55.6%), cognitive‐behavioral therapy (36.1%), and acceptance and commitment therapy (29.1%), with telephone sessions (83.1%) being the most frequently used modality.

### 3.2 Aim 2. Associations Between Mental Health Referral Status and Age Groups

**FIGURE 2:**
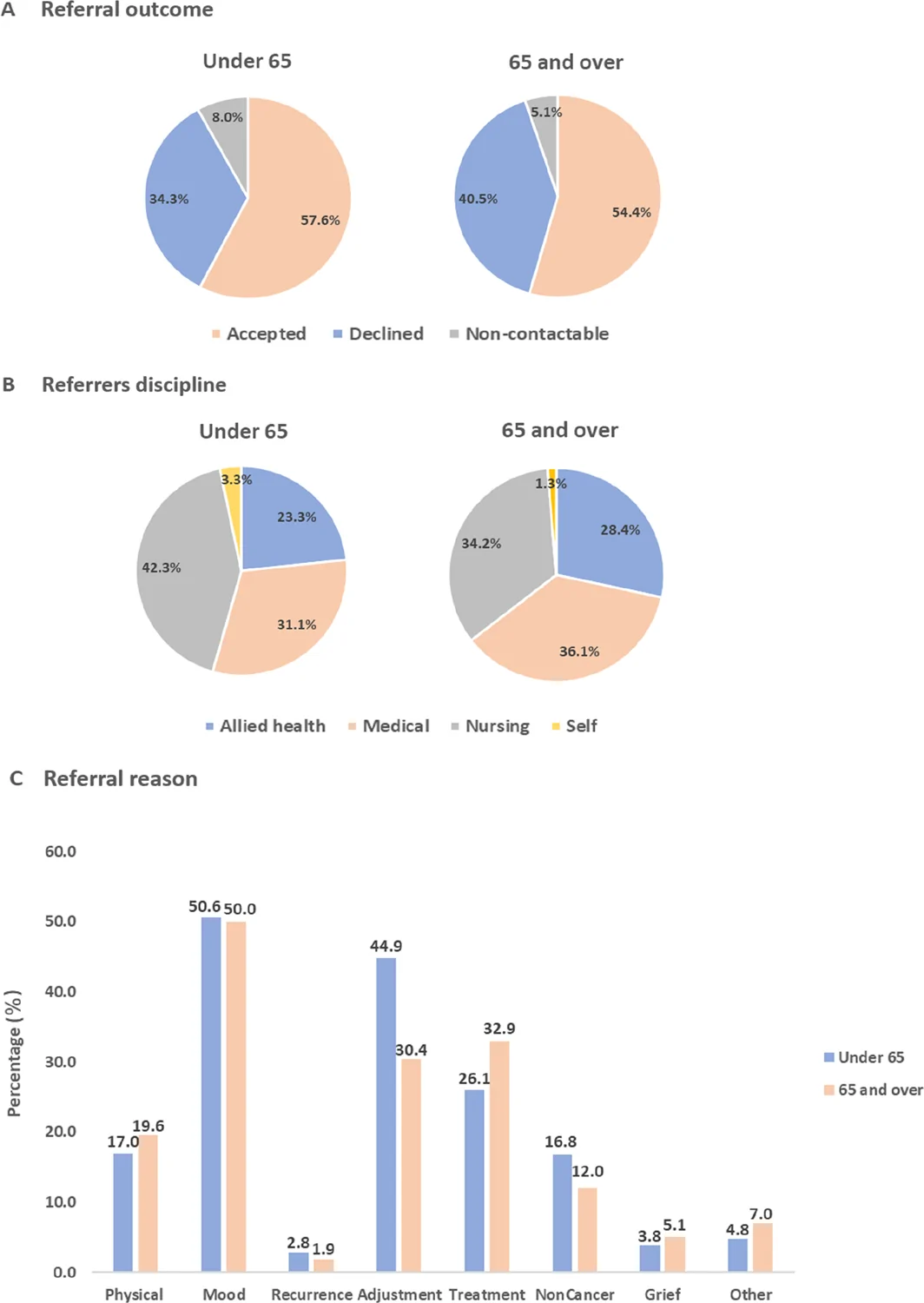
Proportion of referral outcome, referrers discipline and referral reason by age group.

Among the adults with cancer at Peter Mac, older adults accounted for 52.5% but accounted for only 28.4% of the referrals to the Clinical Psychology Department. A one‐sample proportion test indicated that adults with cancer under 65 years of age were more frequently referred to mental health services than older adults (*p* < 0.001). However, there was no difference in referral acceptance or outcomes between the two age groups (*p* = 0.25; See Figure 2A Referral outcome). Chi‐square tests showed that referrer discipline and age group were not associated (*p* = 0.13). Nurses (42.4%) were the most common referral source for adults under 65, while medical staff (36.1%) were the most common source for older adults. Self‐referral was the least common source in both age groups (See Figure 2B Referrers discipline). In both age groups, mood was the reason for referral in 50% of cases (See Figure 2C Referral reason). All associations between referral reasons and age groups were non‐significant (all *p* > 0.13), except for adjustment to cancer (*p* = 0.002), indicating that older adults were less likely to be referred for adjustment compared to those under 65 years.

### 3.3 Aim 3. Potential Reasons Influencing Health Professionals' Referral Decisions

Eighteen health professionals completed the survey. Their characteristics are in Supporting Information S1: Table S8. Most were female (77.8%), aged between 30 and 49 years (66.6%), had over 15 years of work experience (61.1%), and worked in allied health (38.9%) or nursing (27.7%), with 50.0% employed in outpatient settings.

**FIGURE 3:**
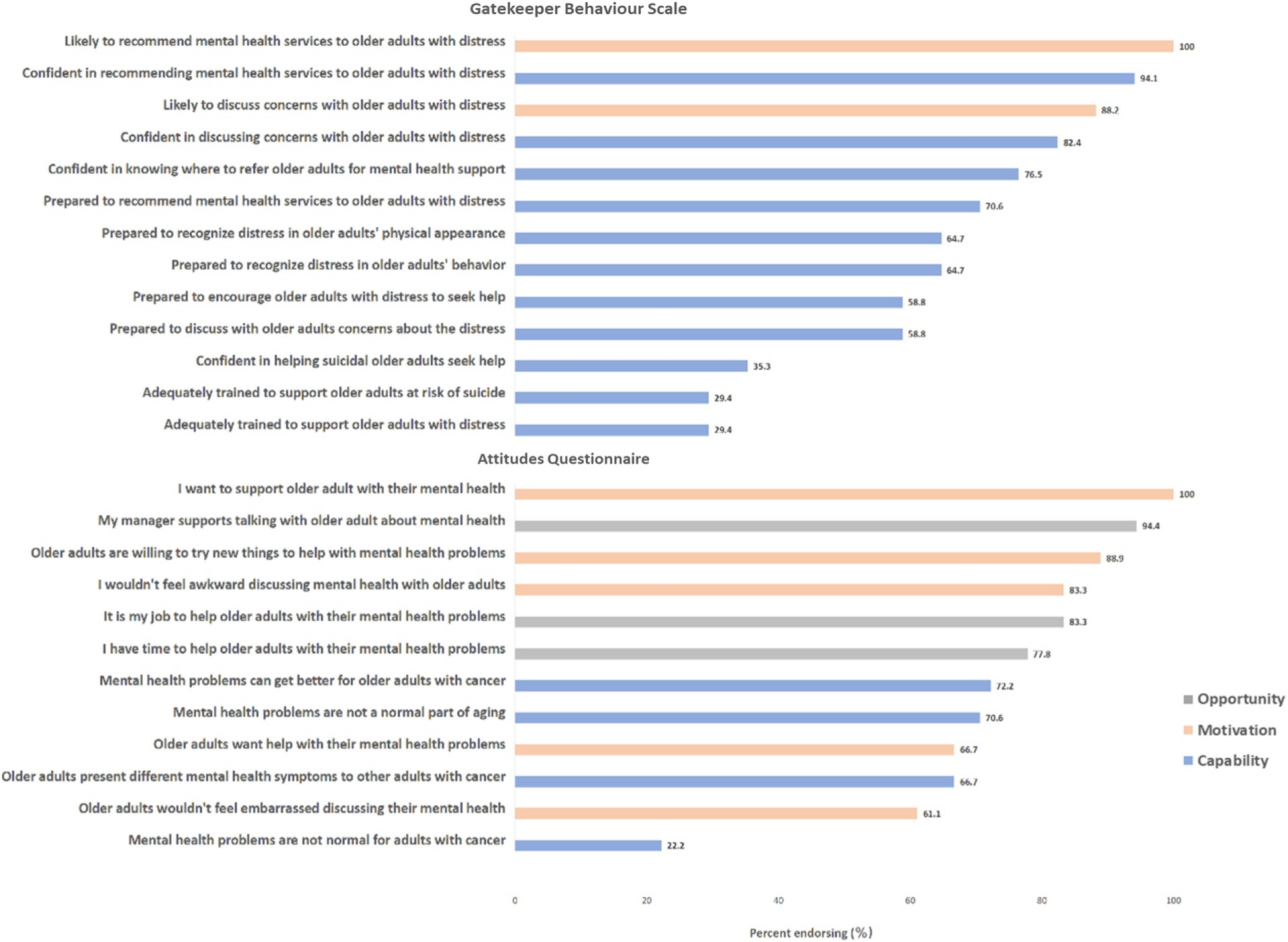
Frequency of gatekeeper behavior scale and attitude questions.

Figure 3 shows responses to the Gatekeeper Behavior Scale and the Attitude Questions. The full text of each item was summarized for clarity. Within the Attitude Questions, capability items showed the lowest proportion of positive responses among healthcare professionals. For nearly half of the capability items, fewer than 60% of healthcare professionals selected the positive response option, indicating potential capability barriers in meeting older adults' mental health needs. The total score of the Gatekeeper Behavior Scale was 42.34 ± 9.71. Although healthcare professionals exhibited high confidence in recommending mental health services to older adults with cancer, discussing issues with patients experiencing psychological distress, and referring them to appropriate psychological support resources, over 70% of healthcare professionals believe they have not received sufficient training to support older adults with cancer in dealing with psychological distress and suicidal tendencies. Additionally, while 70.6% of healthcare professionals agree that mental health issues are not a normal part of the aging process, only 23% agree that poor mental health in cancer patients is abnormal.

Over 75% of healthcare professionals endorsed all the opportunity items. Over 90% of them believe that their manager would not object to them spending time discussing mental health issues, and over 75% indicate that assisting older patients with their mental health problems is part of their job and they have the time to do so. Simultaneously, over 60% of healthcare professionals endorsed all motivation items. The total score of the Mental Health Literacy Scale was 42.22 ± 3.81, indicating a relatively high level. Except for the items “If I had a mental illness, I would tell anyone” and “Adults with a mental illness are not dangerous,” which had an agreement rate of 83.3%, more than 94.4% of healthcare professionals agreed with all the items on the mental health literacy scale. However, more than 30% of healthcare professionals believe that older patients feel embarrassed discussing their mental health issues and are unwilling to accept assistance.

**FIGURE 4:**
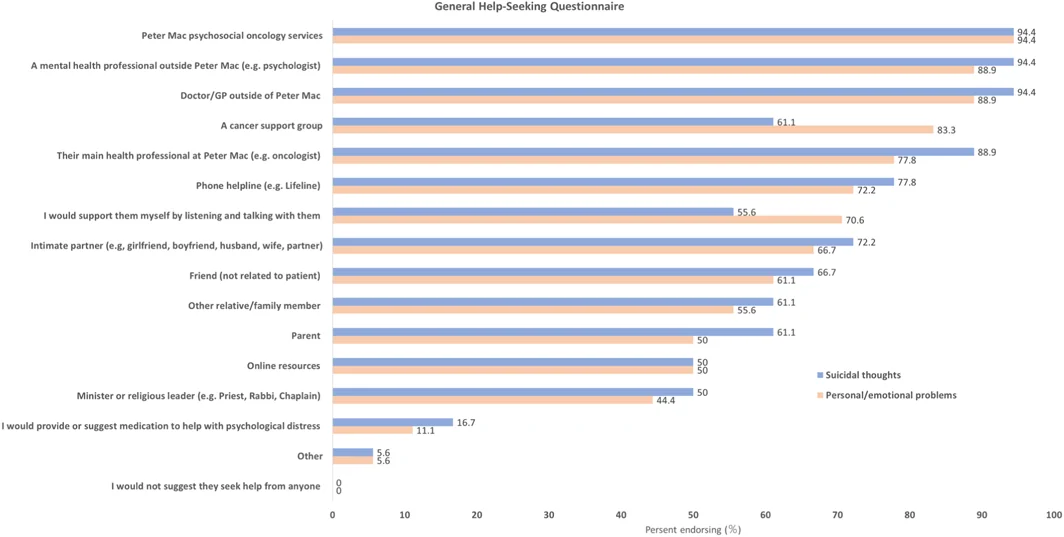
General help‐seeker questionnaire frequencies.

Figure 4 shows the frequency of health professionals' responses to the General Help‐Seeker Questionnaire. Whether patients are facing personal/emotional problems or suicidal thoughts, health professionals most commonly recommend seeking help from Peter Mac psychosocial oncology services, mental health professionals outside of Peter Mac, and doctors outside of Peter Mac. The least endorsed options are medication therapy and providing no assistance at all.

## 4 Discussion

This study shows that older adults with cancer over the age of 65 are referred to mental health services less frequently than those under 65 years old. This is consistent with previous research findings that show a gradual decrease in referrals for psychotherapy as age increases [24]. The reasons for this outcome can be explained as follows. Firstly, older adults' own reluctance towards referral may influence healthcare professionals' inclination to refer them [25]. Research has found that older adults prefer to handle issues themselves [26, 27], and simultaneously hold negative attitudes towards mental health problems, which may lead to a pervasive sense of shame about service referrals and utilization [26, 28]. These attitudes could contribute to healthcare professionals forming stereotypical impressions of older adults. Consistent with the results of this study, nearly 30% of healthcare professionals hold the belief that older adults with cancer feel embarrassed discussing mental health issues and may be reluctant to seek help. This perception can reduce their motivation to refer older adults with cancer.

Disparities may also be related to healthcare professionals' abilities or knowledge, such as believing that psychotherapy is not suitable for older adults or not recognizing their mental health issues, resulting in a lack of referrals [29]. This may be due to a lack of sufficient mental health education and training among healthcare professionals, which was identified as the primary potential barrier to referrals in this study following surveys of healthcare professionals. This finding has been supported by prior research [30, 31]. Healthcare professionals who received targeted mental health training are more likely to discuss mental health issues with patients and refer them for treatment [32]. Although healthcare professionals receive mental health education in schools, Australian mental health academics believe that the current mental health content, theory, and clinical time are insufficient to prepare graduates for practice [33]. Moreover, the differences in healthcare professionals' understanding and confidence when addressing mental health in older adults with cancer and those at risk of suicide also highlight gaps in their education and training or in the availability of trained professionals integrated into the healthcare system who could fill that role. Although these explanations are plausible, they remain speculative as we did not collect data on individual encounters and healthcare professionals, to clarify whether referrals were offered but declined, or what reasons contributed to decision making in specific encounters.

Despite the differences in referral rates among different age groups, we found that once referrals are provided, there is no statistically significant difference in the proportion of older adults and younger adults with cancer accepting referrals. This suggests that many older adults with cancer are willing to accept services, similar to younger patients, and underscores the importance of focusing on older adults who may need mental health services but might not have received adequate referrals. In fact, there has been ongoing debate regarding whether there are differences in psychological distress levels between younger and older adults with cancer. Some studies even suggest that older adults with cancer may be more prone to depression than their younger counterparts [34] which may indicate a greater need for referrals and support. If older adults receive sufficient referrals, they may benefit more from mental health services compared to younger individuals [24]. A study published in 2017 analyzed 82,513 treatment episodes in the South West of England and found that referral rates were higher among young adults. However, once referred, the percentage of individuals achieving significant improvement in treatment gradually increased from 37.08% in the 20–24 age group to 46.17% in the 65–69 age group [24].

Future research should explore feasible and sustainable strategies to increase mental health referral rates for older adults with cancer and evaluate whether such efforts translate into mental health benefits for older adults. For example, professionals might try to proactively discuss mental health with older adults with cancer, overcome their biases, and deepen their understanding of the potential benefits of mental health services.

Our study also found statistically significant differences between the two age groups in the frequency of referrals for adjustment. This may reflect differences in the coping abilities of patients of different ages when facing a cancer diagnosis, prognosis, or terminal diagnosis. Older adults with cancer may have accumulated a broader range of life experiences and resources, having gone through various difficulties and challenges in life. Therefore, they may be more capable of dealing with the adjustments brought by the illness [35]. In contrast, the impact of cancer on the lives of younger patients may be greater, such as work interruptions and changes in family roles [36], so they need more support in adjusting to the illness.

Additionally, contrary to previous studies [37, 38], most healthcare professionals in our study reported having sufficient time to help older adults with cancer with their mental health issues, possibly due to the supportive work environment at the Peter Mac. Over 90% of professionals agreed that managers support discussions on mental health with patients, highlighting the institution's emphasis on and support for patients' mental health. This may be further supported by the staff‐to‐patient ratio at the Peter Mac being 1:3 to 1:4 [39], which is higher than in many other countries [40]. This may contribute to healthcare professionals having more time to address patients' mental health.

### 4.1 Study Limitations

Although Peter Mac is one of the largest cancer centers in Australia, the generalizability of these results may be limited as the data were collected solely from this site. The first part of our study relies on medical records, which are sometimes inadequately documented by staff, potentially affecting the integrity of the data. However, medical records may also be more generalizable than new research studies as many people opt out of data collection. Additionally, in the health professional survey, desirability effects may bias responses, especially in the motivation items. For example, it may be seen as undesirable to report perceiving mental illness as a sign of weakness, even if that is the attitude one holds. However, given that not all items received 100% endorsement and that the survey was anonymous such that no names or explicit identifiers were collected, it can be reasonably assumed that the survey results reflect people's genuine thoughts. Finally, data collection occurred during the peak of the COVID‐19 winter outbreak, which likely contributed to our low staff response rates and may have led to sample bias, as only staff with sufficient time and motivation completed the survey. Findings on staff should be interpreted with caution pending studies with higher response rates to ensure representative samples of healthcare professionals. Future research could replicate these results over time.

### 4.2 Clinical Implications

This study describes the current state of mental health service referrals for older adults with cancer and the potential reasons for low referral rates. This information can help hospitals understand patients' use of mental health services, identify areas needing improvement, and optimize resource allocation and service provision. Our findings show that there is a notable disparity in referral rates between older and younger adults with cancer and highlight this as a place for improvement. Healthcare professional education should provide more comprehensive mental health education. For existing staff, hospitals should consider professional development that targets mental health training. Proactively inquiring about the mental health of adults with cancer, using mental health Patient‐Reported Outcome Measures (PROM) for regular screening, and implementing programs such as an “Enhanced Suicide Prevention Program” may be helpful. The outcomes of education and training should be evaluated and practiced in real‐world applications to ensure effective implementation. Additionally, future research could investigate the opinions and barriers reported by older adults with cancer or examine the actual interactions between patients and healthcare professionals. This would help clarify barriers related to healthcare professionals and related to patients. Finally, our results suggest that hospitals develop quality assurance monitoring to ensure equity in care delivery, including referrals, across patient subgroups, including older adults. Of particular concern, our findings showed that only about 30% of healthcare professionals felt confident in managing suicidality or considered that they had received sufficient training. This highlights the need for more comprehensive education and targeted training in suicide risk assessment and management.

## 5 Conclusions

Compared to younger adults with cancer, the proportion of older adults with cancer referred to mental health services is significantly lower. However, we found that once referred, older adults are just as willing to accept these mental health services as younger patients. Guided by the COM‐B model, we investigated possible reasons for the lower referral rates by healthcare professionals. Capability factors related to training in mental health emerged as the primary barrier. Contrastingly, staff were highly motivated and contrary to some research reported adequate opportunity to support mental health in older adults with cancer.

## Author Contributions

All authors made substantial contributions to this manuscript. The first draft of the manuscript was written by Shuman Wang. Joshua F Wiley gave helpful comments on the manuscript and revised the manuscript. Joshua F Wiley, Maria Ftanou, Lauren Elizabeth Pearson and Chloe Giacomello Nicholas and Kylie King contributed to conception and design. Lauren Elizabeth Pearson and Chloe Giacomello Nicholas contributed to acquisition of data. Shuman Wang and Joshua Wiley contributed to analysis and interpretation of data. All authors reviewed the manuscript.

## Funding

The authors have nothing to report.

## Conflicts of Interest

The authors declare no conflicts of interest.

## Supporting information


Supporting Information S1

## Data Availability

The data underlying this article will be shared on reasonable request to the corresponding author.
