# Supplementary material for: Referral and Utilization of Mental Health Services by Older Adults With Cancer and Factors Influencing Referral From Health Professionals
Source: Psychooncology. 2026 Sep 17;35(9):e70573. doi: 10.1002/pon.70573 (PMC13586635; doi:10.1002/pon.70573)
Supplement: Supplementary file 1 [file PON-35-e70573-s001.docx]

**Figure S1.** Referral Process to the Outpatient Clinical Psychology Department

*Note*. No intake call may have been due to not contacting the individual or not being required.

**Table S1** Gatekeeper Behavior Scale (GBS)

| **Gatekeeper Behavior Scale** | 1 Very Low | 2 Low | 3 Medium | 4 High | 5 Very High |
| --- | --- | --- | --- | --- | --- |
| Recognize when older patient's behavior is a sign of psychological distress |  |  |  |  |  |
| Recognize when older patient's physical appearance is a sign of psychological distress |  |  |  |  |  |
| Discuss with older patient your concern about the signs of psychological distress they are exhibiting |  |  |  |  |  |
| Motivate older patients exhibiting signs of psychological stress to seek help |  |  |  |  |  |
| Recommend mental health support services (such as the counseling center) to older patient exhibiting signs of psychological distress |  |  |  |  |  |
|  | 1 Very Unlikely | | 2 Unlikely | 3 Likely | 4 Very Likely |
| How likely are you to discuss your concerns with older patient exhibiting signs of psychological distress |  | |  |  |  |
| How likely are you to recommend mental health/ support services to older patient exhibiting signs of psychological distress |  | |  |  |  |
|  | 1 Strongly Disagree | | 2 Disagree | 3 Agree | 4 Strongly Agree |
| I feel confident in my ability to discuss my concern with older patient exhibiting signs of psychological distress |  | |  |  |  |
| I feel confident in my ability to recommend mental health support services to older patient exhibiting signs of psychological distress |  | |  |  |  |
| I feel confident that I know where to refer older patient for mental health support |  | |  |  |  |
| I feel confident in my ability to respond to and support older patient who I think might be at risk of suicide |  | |  |  |  |
| I have received adequate training to support older patients who are exhibiting signs of psychological distress |  | |  |  |  |
| I have received adequate training to support older patients who are at risk of suicide |  | |  |  |  |

**Table S2** Mental Health Literacy Scale (MHLS)

|  | 1-Strongly Disagree | 2 -Disagree | 3-Neither Agree or Disagree | 4 -Agree | 5-Strongly Agree |
| --- | --- | --- | --- | --- | --- |
| People with a mental illness could snap out of it if they wanted |  |  |  |  |  |
| A mental illness is a sign of personal weakness |  |  |  |  |  |
| A mental illness is not a real medical illness |  |  |  |  |  |
| People with a mental illness are dangerous |  |  |  |  |  |
| It is best to avoid people with a mental illness so that you don't develop this problem |  |  |  |  |  |
| If I had a mental illness I would not tell anyone |  |  |  |  |  |
| Seeing a mental health professional means you are not strong enough to manage your own difficulties |  |  |  |  |  |
| If I had a mental illness, I would not seek help from a mental health professional |  |  |  |  |  |
| I believe treatment for a mental illness, provided by a mental health professional, would not be effective |  |  |  |  |  |

**Table S3** Attitude Questions

|  | 1 - Definitely  False | 2 - Partially  False | 3- Neutral | 4- Partially  True | 5- Definitely  True |
| --- | --- | --- | --- | --- | --- |
| I want to support older patients with their mental health |  |  |  |  |  |
| Older patients present different mental health symptoms to other cancer patients |  |  |  |  |  |
| Mental health problems are a normal part of ageing |  |  |  |  |  |
| Mental health problems can get better for older patients with cancer |  |  |  |  |  |
| Mental health problems are normal for people with cancer |  |  |  |  |  |
| My manager would not like it if I took time to talk with older patients about their mental health |  |  |  |  |  |
| Older patients do not want help with their mental health problems |  |  |  |  |  |
| Older patients are not willing to try new things to help with mental health problems |  |  |  |  |  |
| Older patients would be embarrassed if I talked with them about their mental health problems |  |  |  |  |  |
| I would feel awkward if I talked with older patients with their mental health problems |  |  |  |  |  |
| It’s not my job to help older patients with their mental health problems |  |  |  |  |  |
| I do not have any time to help older patients with their mental health problems |  |  |  |  |  |

**Table S4** General Help-Seeking Questionnaire (GHSQ)

If older patient was having a personal or emotional problem, how likely is it that you would suggest they seek help from the following people or services?

|  | 1 extremely unlikely | 2 | 3 | 4 | 5 | 6 | 7 extremely likely |
| --- | --- | --- | --- | --- | --- | --- | --- |
| Intimate partner (e.g., girlfriend, boyfriend, husband, wife, partner) |  |  |  |  |  |  |  |
| Friend (not related to patient) |  |  |  |  |  |  |  |
| Parent |  |  |  |  |  |  |  |
| Other relative/family member |  |  |  |  |  |  |  |
| Peter Mac psychosocial oncology services |  |  |  |  |  |  |  |
| A mental health professional outside Peter Mac (e.g. psychologist, social worker, counsellor) |  |  |  |  |  |  |  |
| Phone helpline (e.g. Lifeline) |  |  |  |  |  |  |  |
| Their main health professional at Peter Mac (e.g. oncologist) |  |  |  |  |  |  |  |
| Doctor/GP outside of Peter Mac |  |  |  |  |  |  |  |
| A cancer support group |  |  |  |  |  |  |  |
| Online resources |  |  |  |  |  |  |  |
| Minister or religious leader (e.g. Priest, Rabbi, Chaplain) |  |  |  |  |  |  |  |
| I would not suggest they seek help from anyone |  |  |  |  |  |  |  |
| I would provide or suggest medication to help with psychological distress |  |  |  |  |  |  |  |
| I would support them myself by listening and talking with them |  |  |  |  |  |  |  |
| Other, please describe… |  |  |  |  |  |  |  |

If older patient was experiencing suicide risk, how likely is it that you would suggest they seek help from the following people or services?

|  | 1 extremely unlikely | 2 | 3 | 4 | 5 | 6 | 7 extremely likely |
| --- | --- | --- | --- | --- | --- | --- | --- |
| Intimate partner (e.g., girlfriend, boyfriend, husband, wife, partner) |  |  |  |  |  |  |  |
| Friend (not related to patient) |  |  |  |  |  |  |  |
| Parent |  |  |  |  |  |  |  |
| Other relative/family member |  |  |  |  |  |  |  |
| Peter Mac psychosocial oncology services |  |  |  |  |  |  |  |
| A mental health professional outside Peter Mac (e.g. psychologist, social worker, counsellor) |  |  |  |  |  |  |  |
| Phone helpline (e.g. Lifeline) |  |  |  |  |  |  |  |
| Their main health professional at Peter Mac (e.g. oncologist) |  |  |  |  |  |  |  |
| Doctor/GP outside of Peter Mac |  |  |  |  |  |  |  |
| A cancer support group |  |  |  |  |  |  |  |
| Online resources |  |  |  |  |  |  |  |
| Minister or religious leader (e.g. Priest, Rabbi, Chaplain) |  |  |  |  |  |  |  |
| I would not suggest they seek help from anyone |  |  |  |  |  |  |  |
| I would provide or suggest medication to help with psychological distress |  |  |  |  |  |  |  |
| I would support them myself by listening and talking with them |  |  |  |  |  |  |  |
| Other, please describe… |  |  |  |  |  |  |  |

**Table S5 Coding of questionnaire items**

| Measures | Endorsing Category | Opposing Category | Special Cases |
| --- | --- | --- | --- |
| GBS | very high,  high,  strongly agree,  agree,  very likely,  likely | very low,  low,  strongly disagree, disagree,  very unlikely,  unlikely,  medium |  |
| MHLS | strongly disagree, disagree | strongly agree,  agree,  neither agree nor disagree | all items reversed in content |
| Attitude Questions | definitely false,  partially false | definitely true,  partially true  neutral | reversed for items 1, 2, and 4 |
| GHSQ | scores 5-7 | scores 1-4 | reversed for item 13 |

**Table S6 Demographic characteristics of** **referral, stratified by age (N = 557)**

| Variable | All patients N (%) | < 65 years N (%) | ≥ 65 years N (%) |
| --- | --- | --- | --- |
| Sex |  |  |  |
| Female | 344 (61.8) | 261 (65.4) | 83 (52.5) |
| Male | 212 (38.1) | 137 (34.3) | 75 (47.5) |
| Other | 1 (0.2) | 1 (0.3) | 0 (0.0) |
| Marital Status |  |  |  |
| Partner | 339 (60.9) | 236 (59.1) | 103 (65.2) |
| No Partner | 208 (37.3) | 153 (38.3) | 55 (34.8) |
| Unknown | 10 (1.8) | 10 (2.5) | 0 (0.0) |
| Indigenous status |  |  |  |
| Non- Indigenous | 553 (99.3) | 395 (99.0) | 158 (100.0) |
| Indigenous | 1 (0.2) | 1 (0.3) | 0 (0.0) |
| Unknown | 3 (0.5) | 3 (0.7) | 0 (0.0) |
| Preferred language |  |  |  |
| English | 539 (96.8) | 386 (96.7) | 153 (96.8) |
| Other | 17 (3.1) | 12 (3.0) | 5 (3.2) |
| Unknown | 1 (0.2) | 1 (0.3) | 0 (0.0) |

*Note*. Male and female are biological sexes, not genders. Partner included those who identified as being married or in a defacto relationship. No partner included those who identified as widowed, separated, or single. Unknown means we can't get the information in the electronic medical record system.

**Table S7 Clinical and mental health service characteristics of referral for adults over 65 years**

**(N = 158)**

| Variable | ≥ 65 years N (%) |
| --- | --- |
| Cancer Type |  |
| Gastrointestinal | 28(17.7) |
| Blood | 26 (16.5) |
| Head and neck | 25 (15.8) |
| Genitourinary | 15 (9.5) |
| Breast | 12 (7.6) |
| Skin | 11 (7.0) |
| Lung | 10 (6.3) |
| Gynecological | 10 (6.3) |
| Neuroendocrine | 4 (2.5) |
| Others | 14 (8.9) |
| Unknown | 3 (1.9) |
| Treatment intent |  |
| Curative | 128 (81.0) |
| Palliative | 26 (16.5) |
| Unknown | 4(2.5) |
| Cancer treatment |  |
| Surgery |  |
| Yes | 46 (29.1) |
| No | 93 (58.9) |
| Unknown | 19 (12.0) |
| Radiation therapy |  |
| Yes | 47 (29.7) |
| No | 92 (58.3) |
| Unknown | 19 (12.0) |
| Chemotherapy |  |
| Yes | 53 (33.5) |
| No | 86 (54.5) |
| Unknown | 19 (12.0) |
| Immunotherapy |  |
| Yes | 20 (12.7) |
| No | 119 (75.3) |
| Unknown | 19 (12.0) |
| Targeted therapy |  |
| Yes | 10 (6.3) |
| No | 129 (81.7) |
| Unknown | 19 (12.0) |
| Hormonal therapy |  |
| Yes | 2 (1.3) |
| No | 137 (86.7) |
| Unknown | 19 (12.0) |
| Others therapy |  |
| Yes | 10 (6.3) |
| No | 129 (81.7) |
| Unknown | 19 (12.0) |
| Intake call |  |
| Yes | 132 (83.6) |
| No | 25 (15.8) |
| Unknown | 1 (0.6) |
| Psychological assessment |  |
| Yes | 77 (48.7) |
| No | 80 (50.7) |
| Unknown | 1 (0.6) |
| Psychological care |  |
| Yes | 71 (44.9) |
| No | 78 (49.4) |
| Unknown | 9 (5.7) |
| Number of psychological sessions |  |
| 0 | 34 (21.5) |
| ≤4 | 104 (65.8) |
| ＞4 | 20 (12.7) |
| Support Care Use |  |
| Yes | 97 (61.4) |
| No | 58 (36.7) |
| Unknown | 3 (1.9) |

*Note*. Unknown means we can't get the information in the electronic medical record system.

**Table S8 Characteristics of health professionals (N = 18)**

| Variable | N (%) |
| --- | --- |
| Gender |  |
| Female | 14 (77.8) |
| Male | 4 (22.2) |
| Age |  |
| 18-29 | 2 (11.1) |
| 30-39 | 5 (27.7) |
| 40-49 | 7 (38.9) |
| 50-59 | 3 (16.7) |
| 60-69 | 1 (5.6) |
| Profession |  |
| Allied Health | 7 (38.9) |
| Nursing | 5 (27.7) |
| Medical Staff | 3 (16.7) |
| Other | 3 (16.7) |
| Years working |  |
| ＜6 | 1 (5.6) |
| 6-10 | 4 (22.2) |
| 11-15 | 2 (11.1) |
| >15 | 11 (61.1) |
| Role |  |
| Clinical Care | 13 (72.1) |
| Non-clinical care | 0 (0.0) |
| Management | 3 (16.7) |
| Administration | 1 (5.6) |
| Other | 1 (5.6) |
| Work setting |  |
| Outpatient | 9 (50.0) |
| Inpatient | 2 (11.1) |
| Both | 6 (33.3) |
| Other | 1 (5.6) |
